# NKp44-NKp44 Ligand Interactions in the Regulation of Natural Killer Cells and Other Innate Lymphoid Cells in Humans

**DOI:** 10.3389/fimmu.2019.00719

**Published:** 2019-04-09

**Authors:** Monica Parodi, Herman Favoreel, Giovanni Candiano, Silvia Gaggero, Simona Sivori, Maria Cristina Mingari, Lorenzo Moretta, Massimo Vitale, Claudia Cantoni

**Affiliations:** ^1^Immunology Operative Unit, IRCCS Ospedale Policlinico San Martino, Genoa, Italy; ^2^Department of Virology, Parasitology and Immunology, Faculty of Veterinary Medicine, Ghent University, Merelbeke, Belgium; ^3^Laboratory of Molecular Nephrology, IRCCS Istituto Giannina Gaslini, Genoa, Italy; ^4^Department of Experimental Medicine, University of Genoa, Genoa, Italy; ^5^Center of Excellence for Biomedical Research, University of Genoa, Genoa, Italy; ^6^Department of Immunology, IRCCS Ospedale Pediatrico Bambino Gesù, Rome, Italy; ^7^Laboratory of Clinical and Experimental Immunology, IRCCS Istituto Giannina Gaslini, Genoa, Italy

**Keywords:** natural kiiler cells, innate lymphoid cells (ILC), natural cytotoxicity receptors (NCR), NKp44, ligands, tumor immunology, viral infections

## Abstract

Natural Killer (NK) cells are potent cytotoxic cells belonging to the family of Innate Lymphoid Cells (ILCs). Their most characterized effector functions are directed to the control of aberrant cells in the body, including both transformed and virus-infected cells. NK cell-mediated recognition of abnormal cells primarily occurs through receptor-ligand interactions, involving an array of inhibitory and activating NK receptors and different types of ligands expressed on target cells. While most of the receptors have become known over many years, their respective ligands were only defined later and their impressive complexity has only recently become evident. NKp44, a member of Natural Cytotoxicity Receptors (NCRs), is an activating receptor playing a crucial role in most functions exerted by activated NK cells and also by other NKp44^+^ immune cells. The large and heterogeneous panel of NKp44 ligands (NKp44L) now includes surface expressed glycoproteins and proteoglycans, nuclear proteins that can be exposed outside the cell, and molecules that can be either released in the extracellular space or carried in extracellular vesicles. Recent findings have extended our knowledge on the nature of NKp44L to soluble plasma glycoproteins, such as secreted growth factors or extracellular matrix (ECM)-derived glycoproteins. NKp44L are induced upon tumor transformation or viral infection but may also be expressed in normal cells and tissues. In addition, NKp44-NKp44L interactions are involved in the crosstalk between NK cells and different innate and adaptive immune cell types. NKp44 expression in different ILCs located in tissues further extends the potential role of NKp44-NKp44L interactions.

## Introduction

NK cells are cytotoxic Innate Lymphoid Cells (ILCs) that patrol the body and play a crucial role in the defense against viruses and tumors by circulating in peripheral blood (PB) and extravasating into tissues, in particular at the sites of injury (1–8). In addition to recirculating NK cells, specific NK cell subsets reside in different tissues and organs fulfilling unique regulatory functions. Depending on the organ and the local microenvironment, tissue-resident NK (trNK) cells release proangiogenic factors, regulatory cytokines or specific chemokines, and interact with different cell types. Recirculating NK cells, besides killing target cells, can promote inflammation by cytokine and chemokine release and interaction with dendritic cells (DC), monocytes/macrophages, granulocytes, and T cells (9–14).

NKp44, together with the other Natural Cytotoxicity Receptors (NCRs), NKp46 and NKp30 (14–16) can play an important role in most functions exerted by NK cells and also by other immune cell types (17). Indeed, NKp44 expression is wider than initially thought, and includes activated PB-NK cells (15), interferon-producing intraepithelial ILC1 in tonsils (18, 19), a subset of ILC3 in the decidua (20), and in mucosal-associated lymphoid tissue (MALT) (21), a small subset of decidual trNK cells (22), and plasmacytoid dendritic cells (pDC) (23, 24). Typically, NKp44 is implicated in the killing of virus-infected or tumor cells; however, the increasing panel of NKp44^+^ cells and the identification of new NKp44-ligands (NKp44L), possibly expressed in different tissues or released in the circulation, supports an important role for this receptor in tissue homeostasis and immune regulation (25–29).

NKp44 is a transmembrane glycoprotein characterized by a single extracellular V-type Ig-like domain and a cytoplasmic tail containing an Immunoreceptor Tyrosine-based Inhibitory Motif (ITIM) and no known activating signaling motifs (16). The crystal structure of the NKp44 Ig-V domain reveals the presence of a prominent positively charged groove, likely involved in recognition of anionic patterns shared by different ligands (30). The transmembrane domain contains a charged amino acid (Lys) that allows the association with the Immunoreceptor Tyrosine-based Activating Motif (ITAM)-containing KARAP/DAP12 adaptor protein, a strong transducer of activating signals (16, 31–33). Even if the NKp44 ITIM sequence was originally shown to be non-functional (34), in certain contexts NKp44 can also transduce inhibitory signals. Indeed, NKp44 expressed on pDC inhibits IFN-α release induced upon TLR stimulation (23). More recently, three NKp44 mRNA splice variants (NKp44-1,−2,−3) have been demonstrated to display different signaling capabilities based on the presence (NKp44-1) or absence (NKp44-2 and−3) of the ITIM in their cytoplasmic tail. Local physiologic or pathologic cytokine milieus could influence NKp44 splicing, determining the relative expression levels of these variants and, accordingly, affecting the functional features of NK cells in different locations (see below) (35–37).

The local microenvironment can also determine the function and role of NKp44 by orchestrating development, recruitment, and modulation of specific regulatory, tissue-remodeling, or cytotoxic NKp44^+^ cell subsets (either NK cells or ILCs). Thus, for example, lungs contain a large majority of CD56^dim^ NK cells recirculating from blood; spleen and liver also include CD56^bright^ trNK cells; lymphoid tissues associated to epithelia include NKp44^+^ ILCs; uterus comprises NKp44^+^ NK cells and NKp44^+^ ILC3 with peculiar regulatory and tissue-remodeling functions (2, 4, 5, 18, 19, 38, 39).

Pathologic conditions, and in particular tumors, can result in altered patterns of NKp44^+^ cells. Tumor tissues can produce specific cytokines and chemokines that can drive recruitment of cytokine-producing or cytotoxic NK cell subsets (i.e., CD56^bright^ or CD56^dim^ cells) (40, 41) and induce NKp44 expression. In addition, NKp44^+^ ILC3 have also been described in lung tumors and have been found to be associated with a better clinical outcome (39). On the other hand, in the tumor microenvironment, hypoxic conditions (42), or soluble mediators such as PGE_2_ (43–45) and TGF-β (46) can induce NK cells to down-regulate expression and/or function of major activating NK receptors including NKp44. Similar effects can also be induced by tumor cells and tumor-associated fibroblasts (45, 47).

Thus, depending on the type of available NKp44L, NKp44-expressing cells, tissues, or environmental conditions, the role of NKp44 greatly varies and extends to novel functional contexts, beyond the classical NK-mediated target cell killing.

## NKp44-NKp44L Interactions Involved in Recognition of Tumor and Virus-Infected Cells

### NKp44-Mediated Recognition of Tumor Cells

**Figure 1:**
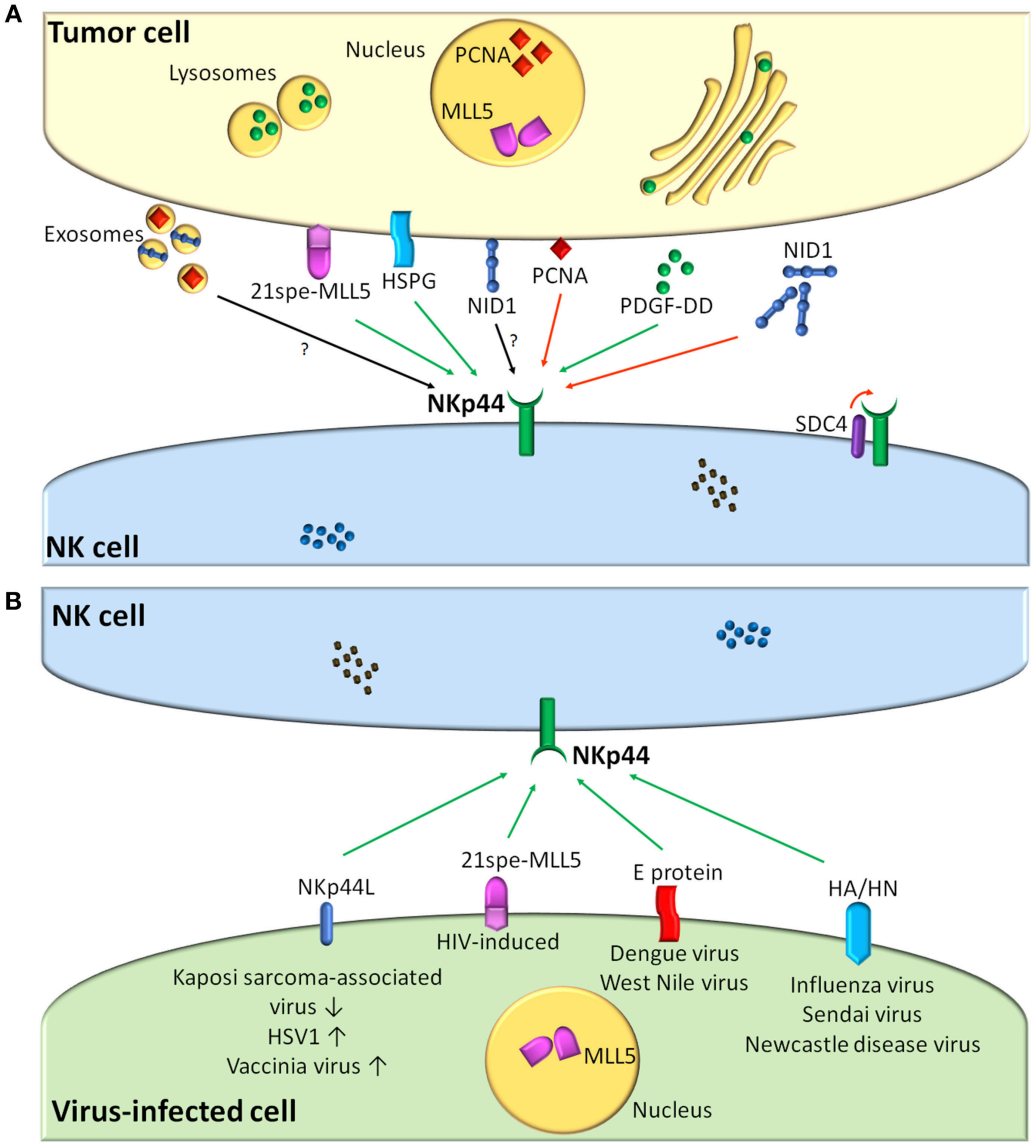
NKp44-NKp44L interactions and their role in the recognition of tumor **(A)** or virus-infected cells **(B)** by human NK cells. **(A)** Shows cellular and cell-released NKp44 ligands expressed by tumor cells. **(B)** Shows viral and viral-regulated NKp44 ligands. Viruses up-regulating/down-regulating NKp44L expression on infected cells are indicated with up and down arrows. Green arrows indicate interactions resulting in NK cell activation, while red arrows indicate inhibition. Black arrows refer to still undefined interactions. MLL5, mixed-lineage leukemia protein-5; PCNA, Proliferating Cell Nuclear Antigen; HSPG, heparan sulfate proteoglycans; NID1, Nidogen-1; PDGF, Platelet-Derived Growth Factor; SDC4, syndecan-4.

Although NKp44L have been elusive for a long time since the discovery of NKp44, experimental evidence suggested that NKp44-NKp44L interactions occurring in the context of tumor cell recognition could play an important role in potentiating NK-mediated cytotoxicity against tumor cells (15, 16, 48, 49). Figure 1A summarizes information on the presently known NKp44-L and their role in the NK-tumor cell interaction.

The first evidence of an activating cellular NKp44L was provided in 2005, when Vielliard et al. reported that an NKp44L is induced on CD4^+^ T lymphocytes from HIV-1-infected individuals (see below) (50). Later on, such NKp44L was identified as a peculiar isoform of mixed-lineage leukemia protein-5 (MLL5), termed 21spe-MLL5, encoded by a splice variant lacking the last five exons of MLL5 and containing a unique C-terminal exon, required for both localization at the cell surface and interaction with NKp44 (51). MLL5 (or lysine methyltransferase 2E), is primarily a nuclear protein with a wide expression pattern in healthy tissues and plays a role in regulating cell cycle progression and hematopoietic differentiation; on the other hand, 21spe-MLL5 is mainly found in the cytoplasm and at the cell surface, is barely expressed in healthy tissues but is frequently detected at high levels in hematopoietic and non-hematopoietic tumor cells. The mechanism allowing the expression of this ligand on the surface of cancer cells is still to be defined, but 21spe-MLL5 surface expression on tumor cells triggers NKp44-mediated cytotoxicity (51).

Another cellular NKp44L is represented by Proliferating Cell Nuclear Antigen (PCNA) (52), a ubiquitously expressed nuclear protein involved in the regulation of DNA replication, DNA repair, and cell cycle progression. Interestingly, it may be overexpressed in cancer cells where it contributes to tumor survival and enhanced malignancy (53). PCNA can be recruited to the plasma membrane of tumor cells, probably through the formation of a PCNA/HLA-I complex (54) or shuttling via exosomes (52). NKp44 engagement by PCNA results in the inhibition of NK-mediated cytotoxicity and IFN-γ secretion by NK cells; this inhibitory effect is mediated by the ITIM in NKp44 cytoplasmic tail and is observed only following an interaction with the ITIM-containing isoform NKp44-1, whose expression has been associated with poor survival in AML patients (36). In view of these findings, PCNA has been proposed as an immune evasion mechanism, enhancing tumor cell survival by promoting a paradoxical NKp44-mediated inhibition of tumor cell killing by NK cells. Recently, the NKp44 binding site for PCNA was identified and an NKp44-derived peptide has been shown to target intracellular PCNA, resulting in the apoptosis of cancer cell lines *in vitro* and tumor growth arrest *in vivo* (55).

Cell surface-associated heparan sulfate (HS) proteoglycans (HSPGs) represent a peculiar category of NCR ligands (56). The three NCRs display a distinct pattern of HS/heparin recognition, based on the heterogeneity and structural complexity of these macromolecules (57). NKp44 recognizes highly sulfated HS/heparin-type structures by binding to negatively charged stretches of HS. Mutations of basic residues in the positively charged NKp44 groove resulted in a decreased binding to HS/heparin. In the context of tumor cell recognition, NKp44 may bind to HS expressed on different cancer cell lines. Moreover, HS was able to enhance NKp44-induced IFN-γ secretion, while the role of HS in the induction of NK-mediated cytotoxicity is less clear (58). Although membrane-associated HSPGs are present on all cells, their expression is heterogeneous in different tissues and can be altered in tumor cells (59). Modified levels of HS in cancer cells may result in altered recognition, in their association with other ligands, or in their structural alterations by tumor-induced modifying enzymes. In this context, HS moieties of HSPGs may be considered as self “modified” ligands for NCRs and may serve as co-ligands, cooperating with other ligands to influence NK cell functions.

NKp44 has also been shown to interact in *cis* with syndecan-4 (SDC4), one of the HSPGs expressed on the surface of NK cells, thereby constitutively dampening NKp44-mediated activation by preventing the receptor binding to other ligands expressed on target cells (60).

More recently, the search for glycolipid ligands by microarray screening led to the identification of Globo-A (GalNAcα1,3(Fucα1,2) Galβ1,3GalNAcβ1,3Galα1,4Galβ1,4Galβ1-Cer) as NKp44L (61). This glycolipid, which was originally isolated from human kidney, displays a globo-series structure and includes a terminal part similar to that of blood group A antigen (62). At present, its functional relevance in the regulation of NK cell function has not been demonstrated yet.

### NKp44-Mediated Recognition of Virus-Infected Cells

Concerning the role of NKp44 in the context of virus recognition, roughly three types of viral interactions have been described: viral NKp44L, virus-induced up-regulation of cellular NKp44L, and virus-mediated inhibition of NKp44 recognition (Figure 1B).

In 2001, Mandelboim et al. reported that the hemagglutinin (HA) of the orthomyxovirus H1N1 influenza virus and the hemagglutinin-neuraminidase (HN) of the paramyxovirus Sendai virus, both expressed on the surface of infected cells, are recognized by NKp46, and thereby trigger the lysis of infected cells by NK cells (63). Shortly thereafter, these viral proteins were also found to serve as NKp44L, but not NKp30L, and the interaction with NKp44 could contribute to the killing activity of certain NK cell clones (64). NKp44 not only recognizes the influenza virus HA of H1 strains but also of H5 strains (65). In addition, HN of other paramyxoviruses, avian Newcastle disease virus and human parainfluenza virus 3 (HPIV3), also appear to serve as NKp44L and trigger NK cell activity (66, 67).

The recognition of both HA and HN depends on sialylation of NKp44, similar to that reported for NKp46 (63–65). Remarkably, the E envelope glycoproteins of two flaviviruses, West Nile and Dengue viruses, also bind to NKp44 thereby increasing NK cell activity, but in a sialylation-independent manner (68).

As mentioned above, in 2005, Vieillard et al. reported that a fraction of CD4^+^ T cells from HIV-infected patients, but not from healthy subjects, showed an increased expression of NKp44L (50). The percentage of NKp44L^+^ CD4^+^ T cells was inversely correlated with the CD4^+^ T cell count and correlated with the viral load, suggesting that NKp44L expression may be implicated in T cell depletion and disease course. In addition, the HIV gp41 envelope protein (and its precursor gp160) was found to trigger NKp44L expression and NK-mediated lysis. In particular, a highly conserved peptide in gp41 (NH2-SWSNKS-COOH or 3S) triggers NKp44L expression (50). Another study suggested that a role for virus-induced NKp44L expression in CD4^+^ T cell depletion during HIV infection may be virus strain-dependent (69).

Members of two large DNA virus families, the poxviruses and the herpesviruses, also up-regulate the expression of NKp44L early after infection of host cells. The immediate early ICP0 protein of herpes simplex virus 1 and an unidentified early protein of the poxvirus vaccinia virus up-regulate expression of NKp30L, NKp44L, and NKp46L, thereby triggering NCR-mediated lysis of infected human fibroblast cells (70, 71).

Viral interference with the NKp44-mediated killing of infected cells typically consists in the down-regulation of cellular or viral NKp44L from the cell surface. Remarkably, the same HIV gp41 S3 peptide that triggers NKp44L expression in CD4^+^ T cells, suppresses NKp44L expression in astrocytes. This led the authors to hypothesize that HIV may use this strategy to protect astrocytes from NK cell-mediated killing during HIV infection of the CNS (72). How the same viral peptide exerts such opposing effects on NKp44L expression and NK cell-mediated cytotoxicity in two different cell types is currently unclear.

The viral ORF54-encoded protein of Kaposi's sarcoma-associated herpesvirus, but not that of other human herpesviruses, suppresses the expression of NKp44L during lytic infection. Although this viral protein is a dUTPase, its enzymatic activity is neither necessary nor sufficient to down-regulate NKp44L (73).

Finally, although the influenza virus HA binds NKp44 and NKp46, thereby contributing to NK-mediated lysis of infected cells, the virus can suppress this effect via expression of its NA protein (74, 75). NA displays sialidase activity which may remove the sialic acids from NKp44 and NKp46, thereby interfering with their binding to HA (74, 75). Although the HA-NA-NKp44 interplay may appear paradoxical at first sight, perhaps this should be looked upon from an evolutionary point of view. The highly conserved function of influenza HA is to interact with sialic acids on the cell surface as an essential initial step to enter host cells. It has been hypothesized that NK cells utilize this general HA sialic acid-binding property to recognize the infected cell via NKp46 and NKp44 (75). The NA protein may then serve as a viral countermeasure against these and other unwanted side-effects of the essential sialic acid-binding activity of HA.

## Regulatory and Homeostatic Functions of NKp44-NKp44L Interactions

NKp44 is also expressed on helper ILCs, which typically reside at the epithelial/mucosal surfaces, becoming involved in tissue remodeling, inflammation, and maintenance of barrier integrity (76). Notably, NKp44 expression allows to discriminate two ILC3 subsets characterized by unique cytokine patterns: NKp44^+^ ILC3 are able to produce IL-22 (21) and are dependent on the Aryl Hydrocarbon Receptor, whereas human LTi and NKp44^neg^ ILC3 produce IL-17A (77). Remarkably, in NKp44^+^ ILC3, IL-22 expression is preferentially induced by cytokine stimulation, whereas NKp44 triggering selectively activates a coordinated pro-inflammatory program via TNF-α (78). In this context, NKp44 has been suggested to play a potential role in the pathogenesis of immune-mediated diseases, including psoriasis. Indeed, more than 50% of circulating NKp44^+^ ILC3 in the blood of psoriasis patients express cutaneous lymphocyte-associated antigen, suggesting their role in skin homing. Moreover, NKp44^+^ ILC3 frequency in non-lesional psoriatic skin is significantly increased when compared to that in normal skin (79).

NKp44^+^ ILC3 are also present in secondary lymphoid organs (SLO). Differently from ILC3 in inflamed tonsils, ILC3 in non-inflamed lymph nodes and spleen, irrespective of NKp44 expression, lack the transcription of cytokines typically mediating ILC3 function. However, both NKp44^neg^ and NKp44^+^ resting ILC3 can produce IL-22 in response to inflammatory stimuli. According to these data, SLO-residing ILC3 may represent a pool of resting cells that can be rapidly activated by inflammatory signals present in the local microenvironment (80).

NKp44^+^ ILC3 can also infiltrate tumor tissues. In particular, in non-small cell lung cancer, NKp44 can interact with tumor cells and synergize with IL-1β and IL-23 for IL-22 production (39). In addition, NKp44-mediated recognition of tumor associated fibroblasts can lead to the release of high amounts of TNF-α, thus influencing the vascular permeability and inducing pro-inflammatory responses in the tumor microenvironment (39).

In the uterus, the NKp44^+^ pool is composed of ILC3, LTi-like cells, IFN-γ-producing ILC1-like cells, and NK cells (20, 22). These cell populations are variably involved in tissue remodeling related to decidua development and vascularization during pregnancy, the induction of maternal-fetal tolerance, and the control of viral infections (i.e., CMV) (81). For some of these functions the role of NKp44 has been suggested by its ability to induce IP10, IL-8, and VEGF release (82). It has also been recently shown that the decidual cytokine milieu can favor the expression of the ITIM-bearing NKp44-1 inhibitory isoform (37), which indeed can induce cytokine release but inhibits cytotoxicity. In this context, the overexpression of the NKp44-L PCNA in decidual throphoblasts in the first trimester of gestation suggests that inhibitory signaling of NKp44 might contribute to the maintenance of immune tolerance of maternal NK cells during pregnancy (83).

As mentioned in the Introduction, the functional role of NKp44 also depends on the nature and body distribution of its ligands. Very recently, the discovery of Platelet-Derived Growth Factor (PDGF)-DD as a novel NKp44L represented a major breakthrough, since for the first time NKp44 was demonstrated to recognize a soluble molecule (28, 84). PDGF-DD belongs to the PDGF family and represents the active processed form of PDGF-D (85, 86). Noteworthy, PDGF-DD-NKp44 interaction does not trigger NK cell-mediated cytotoxicity, rather, it induces potent release of TNF-α and IFN-γ by IL-2-activated NK cells. Transcriptomic analysis clearly indicated that stimulation of activated NK cells by PDGF-DD induced genes encoding pro-inflammatory cytokines and chemokines, cell surface activation markers, and transcription factors involved in ITAM signaling, cellular activation, and proliferation. Notably, the induction of an NKp44-dependent pro-inflammatory program upon PDGF-DD-NKp44 interaction has been demonstrated not only in activated NK cells but also in other NKp44^+^ ILCs, namely ILC3 and intraepithelial ILC1. In particular, PDGF-DD induced IFN-γ and TNF-α production in ILC1, while it stimulated TNF-α but not IL-22 secretion in ILC3 (28).

PDGF-DD is involved in embryonic development, placenta formation, angiogenesis, and wound healing, and has been implicated in various pathological conditions, including vascular diseases, mesangioproliferative glomerulonephritis, and fibrosis (85, 87). Thus, PDGF-DD interaction with NKp44^+^ cells present in various tissues (including decidua) may play diverse biological roles. Nevertheless, PDGF-DD can be secreted by different tumors and promotes cellular proliferation, stromal cell recruitment, angiogenesis, epithelial-mesenchymal transition, and metastasis through autocrine and paracrine PDGFRβ signaling (88, 89). In this context, PDGF-DD, as NKp44L, can also induce NK-, ILC1- and ILC3-mediated release of cytokines with anti-tumor activity. Indeed, supernatants of PDGF-DD-activated NK cells inhibit tumor cell growth *in vitro*, while *in vivo* experiments indicated that the introduction of the NCR2 transgene limited the spread of PDGF-DD-expressing tumor cells in mice (28).

The example of PDGF-DD as an extracellular ligand is not unique among NCRs, since NKp46 can also bind to a soluble molecule, namely the plasma glycoprotein Complement Factor P (CFP or properdin) (90). Both CFP and PDGF-DD activate NK cell functions. However, soluble ligands of activating receptors generally represent mechanisms to modulate NK cell functions (91–97). In this context, another peculiar NKp44L was recently described (29). Nidogen-1 (NID1) glycoprotein is an essential component of ECM and basement membrane (BM) (98, 99) able to interact with NKp44. When released as soluble molecule, NID1 modulates NK cell function by reducing NKp44-induced IFN-γ release or cytotoxicity. Notably, it also regulates IFN-γ production induced by PDGF-DD following NKp44 engagement. Thus, the release of NID1 in extracellular fluids may act as regulatory mechanism for NKp44^+^ NK cells in the blood stream or in tissues. Interestingly, NID1 release has been observed in different cancer types (100–102), and may represent a suppressive mechanism exploited by tumors to avoid NK-mediated attack. Actually, the functional outcome of the NKp44-NID1 interaction may be rather complex, as NID1 can associate with other ECM components, including laminin, collagen type IV, and perlecan (98, 99), or be modified by extracellular proteases secreted in the tumor microenvironment (103, 104). In addition, NID1 can also be exposed at the cell surface of different NID1-releasing tumor cell lines (29).

As shown by the examples of PDGF-DD and NID1, NKp44L can be expressed not only on neoplastic or virus-infected cells, but also on normal cells. Thus, NKp44L expression was observed on the surface of articular chondrocytes (105), although the nature of this ligand is not well-defined. Since NK cells can kill human chondrocytes, it has been suggested that the NKp44-NKp44L interaction may play a role in cartilage destruction occurring in chronic inflammatory joint disorders. Interestingly, human NK cells were previously shown to kill porcine chondrocytes mainly through NKp44-NKp44L interaction (106).

More recently, it has been demonstrated that human astrocytes express an NKp44L whose interaction with NKp44 contributes to NK-mediated cytotoxicity against astrocytes and to IFN-γ production (72).

## Concluding Remarks

The finding that NKp44 is expressed by different immune cell types and tissues (Table 1) and can recognize multiple ligands (soluble or associated to the cell surface or ECM) strongly suggests that this receptor is used to fulfill various functions, adapted to different body compartments or even to temporary micro-environmental changes.

**Table 1:** Expression of NKp44 ligands in human tissues.

Ligand (inhib/act NKp44)^*^	**Tissues**
**PCNA**^a^^,^^b^ (inhibition through ITIM)	***Nervous system***: cerebral and prefrontal cortex, spinal cord, cerebellum
	***Endocrine tissues***: adrenal, thyroid, parathyroid, and salivary glands, pancreas
	***BM***^e^ ***and immune system***: B, T, and NK cells, monocytes, BM stromal cells, LN^e^, tonsil, spleen, appendix
	***Muscle tissues***: heart, smooth and skeletal muscle
	***Respiratory system***: lung, nasopharynx, bronchus
	***Skeletal system***: bone
	***Digestive system***: colon and ileum epithelial cells, colon, esophagus, gallbladder, gut, cardia, liver, oral epithelium, stomach, rectum, duodenum, small intestine
	***Urinary system***: kidney, bladder
	***Male tissues***: testis, seminal vesicle, prostate gland, epididymis
	***Female tissues***: breast, amniocytes, fallopian tube, myometrium, ovary, placenta, uterus, uterine cervix, vagina, endometrium
	***Adipose and soft tissues***: adipocytes
	***Integumentary system***: hair follicle, skin
**21spe-MLL5**^c^ (activation)	***BM and immune system***: CD4^+^ T cells from HIV-infected patients
	***Skeletal system***: articular chondrocytes
**Syndecan-4**^a^^,^^b^ (inhibition through receptor masking)	***Nervous system***: cerebral and prefrontal cortex, spinal cord, cerebellum, hippocampus, caudate
	***Endocrine tissues***: adrenal, thyroid, parathyroid, and salivary glands, pancreas
	***BM and immune system***: platelets, LN, tonsil, spleen, appendix
	***Muscle tissues***: heart, smooth and skeletal muscle
	***Respiratory system***: lung, nasopharynx, bronchus
	***Skeletal system***: bone, chondrocytes
	***Digestive system***: colon, gallbladder, liver, oral epithelium, rectum, small intestine, duodenum, stomach, esophagus
	***Urinary system***: kidney, bladder
	***Male tissues***: testis, seminal vesicle, prostate gland, epididymis
	***Female tissues***: breast, amniocytes, fallopian tube, myometrium, ovary, placenta, uterine cervix, vagina, endometrium
	***Adipose and soft tissues***: adipocytes, fibroblasts
	***Integumentary system***: skin
**PDGF-DD**^b^^,^^d^ (activation)	***Nervous system***: cerebral cortex
	***Endocrine tissues***: adrenal and thyroid gland
	***BM and immune system***: LN, tonsil, spleen, appendix
	***Muscle tissues***: heart, smooth muscle
	***Respiratory system***: lung
	***Digestive system***: colon, oral epithelium
	***Male tissues***: seminal vesicle
	***Female tissues***: breast, ovary
	***Integumentary system***: skin
**NID1**^a^^,^^b^ (inhibition through receptor masking)	***Nervous system***: arachnoid cyst, cerebral cortex, prefrontal cortex, spinal cord
	***Endocrine tissues***: adrenal, thyroid, and salivary glands, pancreas
	***BM and immune system***: platelets, mesenchymal stem cells, NK cells, LN, tonsil, spleen
	***Muscle tissues***: colon muscle, heart
	***Respiratory system***: lung, nasopharynx
	***Digestive system***: colon, esophagus, gallbladder, gut, cardia, liver, oral epithelium, stomach, rectum;
	***Urinary system***: kidney, bladder
	***Male tissues***: testis, seminal vesicle, prostate gland, epididymis
	***Female tissues***: breast, amniocyte, myometrium, ovary, placenta, uterus, uterine cervix
	***Adipose and soft tissues***: adipocytes
	***Integumentary system***: skin

* *NKp44-NKp44L interactions can result in a different outcome depending on the ligand: activation or inhibition of NK cell functions*.

a *Data obtained from ProteomicsDB*.

b *data obtained from The Human Protein Atlas*.

c *data obtained from refs (50, 51, 105)*.

d *data obtained from ref (107)*.

e *BM, bone marrow; LN, lymphnodes*.

The expanding knowledge on the multifaceted role of NK cell subsets and ILCs, residing in the different tissues, suggests that NKp44-NKp44L interactions may be involved in the activation/regulation of several biological/immunological processes. A deeper understanding of these issues will be beneficial for the design of NK-based immunotherapeutic approaches in different pathologic conditions, including but not limited to tumors and infections.

## Author Contributions

MP, HF, SS, MV, and CC wrote the manuscript. GC, SG, MCM, and LM reviewed the manuscript and provided critical input.

### Conflict of Interest Statement

The authors declare that the research was conducted in the absence of any commercial or financial relationships that could be construed as a potential conflict of interest.
